# LRRK2 and the fragile synapse: a molecular prelude to Parkinson’s disease?

**DOI:** 10.1042/BCJ20253351

**Published:** 2025-10-28

**Authors:** Beatrice Masotti, Giulia Tombesi, Loukia Parisiadou, Elisa Greggio

**Affiliations:** 1 Department of Biology, University of Padova, Italy; 2 Northwestern University, Chicago, U.S.A.; 3 Multidisciplinary Institute of Ageing, MIA-Portugal, University of Coimbra, Portugal; 4 Centro Studi per la Neurodegenerazione (CESNE), University of Padova, Italy

**Keywords:** Synapses, Phosphorylation, LRRK2, Dopaminergic neurons, Neurodegeneration, Parkinson’s disease

## Abstract

Parkinson’s disease (PD) is a multisystem disorder presenting motor and non-motor symptoms. Motor dysfunction is the most debilitating, caused by the degeneration of dopamine-producing neurons. Increasing evidence indicates that synapse demise occurs years before neuronal death. Yet, the early synaptic dysfunctions in PD remain poorly understood. Leucine-Rich Repeat Kinase 2 (LRRK2), a serine/threonine kinase and GTPase relevant for both familial and sporadic forms of PD, has been increasingly associated with synaptic processes. These include the phosphorylation of key synaptic proteins and interactions with cytoskeletal components. Brain-derived neurotrophic factor (BDNF) and glial-derived neurotrophic factor (GDNF) are fundamental for synapse maturation, maintenance, and plasticity. Recent findings indicate that neurotrophic signaling is impaired in PD. In this review, we critically discuss the significance of identifying and clarifying the early molecular events leading to synaptic dysfunction in PD. We examine how mutant LRRK2 affects these processes and the relationship between LRRK2 and BDNF signaling from both mechanistic and therapeutic perspectives.

## Introduction: Parkinson’s disease and the role of synaptic dysfunction

Over 8.5 million people worldwide are currently living with Parkinson’s disease (PD) (World Health Organization, 2019). This number is expected to more than double, exceeding 17 million by 2040–2050, primarily due to global aging trends [1]. PD can occur as either a hereditary or sporadic condition that progressively impairs the function of dopaminergic neurons of the substantia nigra pars compacta (SNpc), ultimately leading to their loss. Dopaminergic neurons project from the SNpc to the dorsal striatum, a region that serves as an input stage of the basal ganglia, essential for generating and controlling voluntary movements [2]. At later stages, PD also affects other brain regions, causing severe motor, cognitive, affective, and autonomic dysfunctions [2]. Motor dysfunction is particularly debilitating, and it is caused by dopaminergic neuron degeneration. In addition to nigral neuron degeneration, the PD pathology is characterized by the presence in surviving neurons of intracellular aggregates termed Lewy bodies (LBs). LBs are enriched in phosphorylated alpha-synuclein (α-syn), a presynaptic protein that is also mutated in autosomal dominant PD [3,4].

Multiple lines of evidence indicate that, early in the disease process, the loss of dopaminergic axonal terminals in the striatum exceeds the loss of their neuronal cell bodies in the SNpc [5,6]. Neuroimaging studies using dopamine transporter (DAT) ligands have consistently shown that during the early symptomatic stages of PD, the degeneration of dopamine release sites and axonal terminals is more pronounced than that of the cell bodies and proximal axons [7,8]. This pattern indicates that synaptic dysfunction and terminal loss represent the initial pathological events, which may then trigger a ‘dying-back’ process characterized by retrograde axonal degeneration, ultimately leading to neuronal death [9].

Currently, the primary treatment for PD involves the use of L-DOPA or dopamine receptor agonists. While these treatments can alleviate symptoms, they do not halt disease progression and are poorly tolerated in the long run [2]. This creates an urgent need to better understand the molecular mechanisms that lead to PD in order to design effective disease-modifying therapies. Unlike irreversible neuronal cell loss, synaptic dysfunctions are potentially reversible. The nervous system retains the capacity to regenerate synaptic terminals and dendritic spines, offering a window of opportunity for therapeutic intervention [10,11]. Therefore, directing PD research towards early, prodromal synaptic dysfunction, rather than waiting until late-stage neuronal death, is critical to enable the design of effective disease-modifying and preventive strategies that preserve neuronal circuits and delay or halt disease progression. This perspective aligns with well-established findings in Alzheimer’s and Huntington’s diseases, where early synaptic loss precedes clinical symptoms [12,13]. In PD, however, this concept is only now emerging, underscoring the significance and timeliness of focusing on early synaptic pathology.

The etiology of PD is complex and multifactorial, involving the interaction between genetic and environmental factors. Genetics is estimated to represent about 25% of overall PD, reflecting the combined influence of rare monogenic mutations and common genetic variants identified through genome-wide association studies (GWAS) [14]. Approximately 10% of PD cases are caused by mutations in single genes with both dominant (e.g. *SNCA, LRRK2, GBA, VPS35*) and recessive (e.g. *PRKN, PINK1, VPS13C, DNAJC6, SYN1*) patterns of inheritance and variable penetrance [14]. These genetically defined forms of PD present a unique opportunity to dissect the underlying mechanisms of the disease through cellular and animal models [15]. Notably, recessive, early-onset loss-of-function mutations in *SYNJ1* and *DNAJC6* encode the pre-synaptically enriched proteins synaptojanin 1 and auxilin [16,17].

Among the monogenic forms of PD, mutations in the *Leucine-Rich Repeat Kinase 2* (*LRRK2)* gene are one of the most common causes of autosomal dominant PD. Additionally, more frequent non-coding and coding variants in the *LRRK2* gene increase the lifetime risk of developing sporadic PD [18]. Pathogenic *LRRK2* mutations are predominantly clustered in the catalytic Roc-COR GTPase (N1437H, R1441G/C/H, and Y1699C) and kinase (G2019S, I2020T) domains, resulting in increased substrate phosphorylation, particularly of a subset of Rab GTPases, the master regulators of vesicular trafficking [19,20]. Hyperphosphorylation of Rabs, including Rab3a, Rab8a, Rab10, and Rab35, disrupts endolysosomal trafficking, leading to defects in synaptic vesicles (SV) cycling, lysosomal repair mechanisms, and ciliogenesis [20–22].

**Figure 1:**
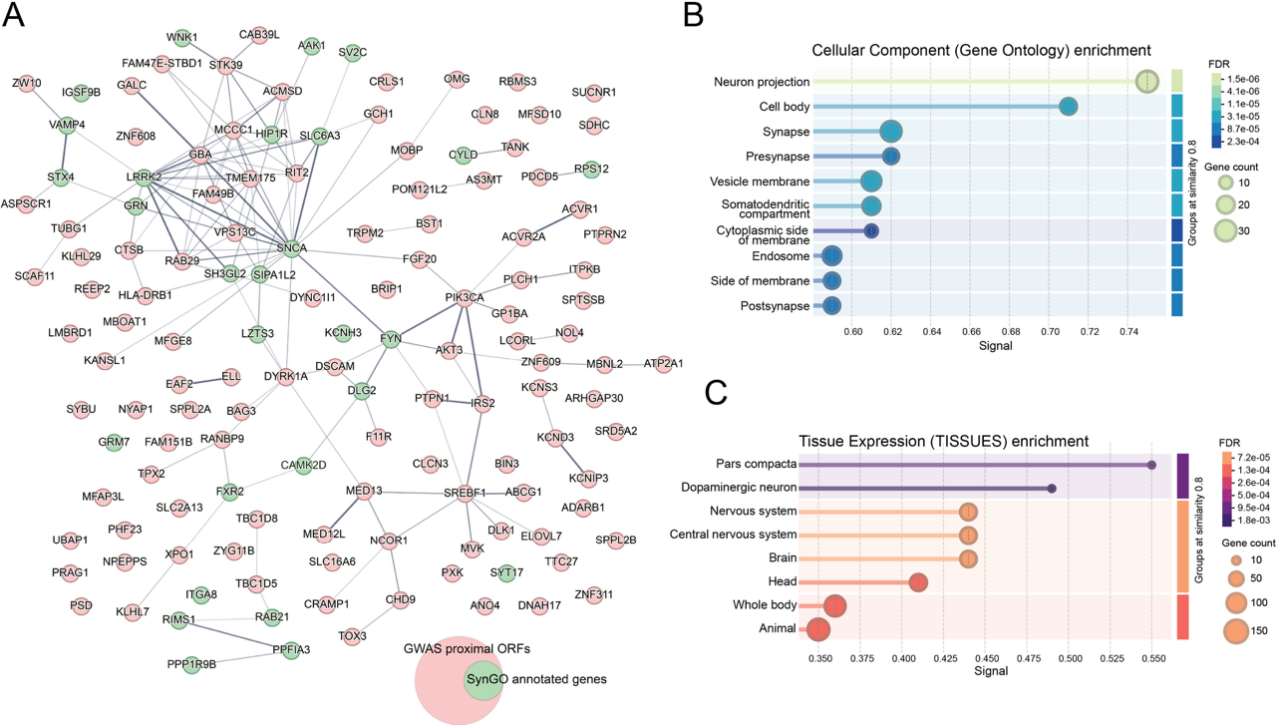
Network and functional enrichment analyses of the ORFs proximal to the identified risk signals in the latest GWAS meta-analysis. Genes were retrieved from online supplementary table 3 of [23]. **A**. Protein–protein interaction (PPI) and enrichment analyses were performed using the STRING database (version accessed on September 19, 2025) [25]. Thirty-two nodes represent synaptic proteins retrieved as SynGO-annotated genes (dataset version 20231201, accessed September 19, 2025) and are highlighted in green. Number of nodes: 137; number of edges: 143; average node degree: 2.09; average local clustering coefficient: 0.406; expected number of edges: 71; PPI enrichment *P*-value: 4.72e-14. **B-C**. STRING enrichment analyses highlight the synaptic and dopaminergic relevance of the gene set. Cellular component enrichment (**B**): the input genes are strongly enriched for neuronal and synaptic compartments, with top categories including neuron projection, cell body, and synapse, as well as presynaptic and vesicle membrane components. Tissue expression enrichment (**C**): the same gene set is most strongly associated with SNpc and dopaminergic neurons, key sites of vulnerability in PD, with additional enrichment in the broader nervous system and brain tissues.

Beyond causal genes, large-scale GWAS have significantly enhanced and expanded our understanding of PD genetics. The latest meta-analyses, including over 63,000 PD cases and more than 1.7 million controls, identified 134 independent risk loci for PD, 59 of which are novel discoveries [23]. This marks a substantial increase from previous GWAS and highlights the polygenic nature of PD [24]. Although GWAS risk loci do not directly identify causal genes, the open reading frames (ORFs) proximal to these identified risk signals are strongly enriched for genes expressed in the SNpc and dopaminergic neurons and involved in synaptic processes (Figure 1 and [23]). Moreover, a protein–protein interaction (PPI) network analysis of 96 genes linked to hereditary parkinsonism identified LRRK2 and α-syn as central hub proteins, with chemical synaptic transmission and vesicle-mediated transport at the synapse among the most significantly enriched functional categories [26]. Together, these observations provide direct genetic evidence that synaptic dysfunction can act as a primary driver of PD pathogenesis, rather than as a downstream consequence of neuronal loss [27].

Although the neuropathological hallmark of PD is typically the presence of LBs enriched in α-syn aggregates, carriers of *LRRK2* G2019S mutation often lack these classical inclusions, raising the question of whether LRRK2-PD is driven by shared or divergent mechanisms and whether it can serve as a faithful model of sporadic PD [28,29]. Recently, two independent studies employing sensitive detection methods like proximity ligation assays revealed widespread early-stage α-syn oligomers in LRRK2-PD brains, even in the absence of mature LBs [30,31]. These novel observations support the concept that LRRK2-PD involves the accumulation of presynaptic oligomeric, non-fibrillar α-syn species, which are recognized as the most neurotoxic forms [32]. Considering the crucial role of LRRK2 in endolysosomal degradative pathways and the impairment of autophagy-lysosomal degradation caused by mutant LRRK2 [33,34], this early-stage α-syn oligomerization may arise from, or be exacerbated by, pathological LRRK2 hyperactivity, contributing to initial synaptic dysfunction. Thus, LRRK2-PD provides a valuable framework for studying early synaptic mechanisms of disease, in which α-syn aggregation remains at its most toxic, pre-LBs phase.

Collectively, these findings support a model where neuron-autonomous synaptic dysfunction occurs early, potentially driven or accelerated by a subset of genetic risk factors that code for proteins with a role at the synapse. Astrocytes, which form the tripartite synapse by enveloping neuronal synapses [35], may sense this dysfunctional synaptic activity [36,37] and amplify pathological signaling, possibly in concert with microglia, the brain’s resident immune cells [38]. Microglia and, to a lesser extent, astrocytes are responsible for the phagocytic removal of compromised synapses [36,37], a process that, if dysregulated, could aggravate synaptic loss and neuronal damage [13].

In the following sections, we examine the hypothesis that pathogenic LRRK2 hyperactivity creates a maladaptive synaptic environment, acting as an initial faulty cog that triggers cascading cellular and network-wide mechanisms leading to PD. We also explore the potential links between LRRK2 pathobiology and neurotrophic factors, particularly brain-derived neurotrophic factor (BDNF), highlighting implications for therapeutic strategies.

## Dopaminergic neuron subtypes and vulnerable synapses: a focus on LRRK2

Dopaminergic neurons of the SNpc that are preferentially lost in PD are uniquely vulnerable due to their distinctive intrinsic properties. These have been comprehensively reviewed in [39,40]. These neurons possess highly branched, unmyelinated axons that can form up to 245,000 synaptic contacts per neuron [9]. This extraordinary complexity of axonal arborization imposes a massive bioenergetic demand, requiring a continuous ATP supply to maintain ionic gradients for action potentials, SV cycling, and neurotransmitter release and uptake. Additionally, the large axonal surface area and extensive synaptic connectivity necessitate efficient protein quality control and organelle maintenance, particularly through autophagic and mitophagy pathways, to prevent accumulation of damaged proteins and dysfunctional mitochondria [41]. Of interest, the observed resilience of mouse dopaminergic neurons to neurodegeneration in the different genetic PD mouse models might be explained by the relatively smaller size of their axonal arborization and consequent lower energetic demand [42,43]. Supporting this, an increased vulnerability of nigral dopaminergic neurons has been observed upon conditional deletion of mouse D2 dopamine receptors, which causes expansion of dopaminergic axonal arborization [44]. However, it is challenging to determine whether the expanded arborization resulting from D2 receptor deletion is solely responsible for the heightened vulnerability or if additional processes related to its loss, such as alterations in dopamine metabolism, also contribute to this effect [45].

Subpopulations expressing high levels of calcium channels (Cav1), low calcium buffering, and relying heavily on autonomous pacemaking activity are particularly susceptible [39]. This is partly due to sustained calcium influx, increasing mitochondrial oxidative stress and reactive oxygen species (ROS) production. Other dopaminergic neurons, such as those projecting to the ventral tegmental area (VTA), display more robust calcium buffering and lower metabolic stress, correlating with relative resistance to degeneration [39]. Of note, Surmeier and colleagues demonstrated that selective disruption of mitochondrial complex I function in dopaminergic neurons induces progressive parkinsonism in mice, emphasizing the critical role of mitochondrial bioenergetics in neuronal survival [46]. Elevated cytosolic calcium due to poor buffering and increased ROS, alongside defective proteostasis, can promote or exacerbate α-syn oligomerization, which collectively compromises synaptic function, leading, in turn, to PD pathology. Thus, the synapses of dopaminergic neurons are likely particularly vulnerable due to their high energy demand for processes such as vesicle recycling and the maintenance of the neurotransmitter release machinery.

Previous studies have mainly focused on the selective vulnerability of dopaminergic neurons in the SNpc, compared with the less vulnerable dopaminergic neurons in the adjacent VTA area [39]. However, recent single-cell transcriptomic studies and anatomical mapping have revealed notable heterogeneity in dopaminergic neurons within the SNpc, complicating our understanding of selective susceptibility in PD. These neuronal subpopulations exhibit distinct gene expression profiles, diverse projection targets and functional properties, and, importantly, a different pattern of vulnerability in PD [47–50]. Research indicates that the ventral tier neurons are lost to a greater extent than the dorsal tier ones [51]. Therefore, understanding the intrinsic properties that contribute to the vulnerability of specific dopaminergic neuron subtypes, along with their associated synaptic dysfunctions, is crucial in explaining why these neurons degenerate preferentially in PD.

Recently, work by Awatramani and collaborators described a high-resolution molecular and spatial map of midbrain dopaminergic neurons, utilizing an advanced single-nucleus transcriptomics pipeline. This refined approach enabled a more granular classification of dopaminergic neurons, revealing even further heterogeneity. The team also focused on analyzing gene expression changes in dopaminergic neurons carrying the murine G2019S mutation [52]. Strikingly, their findings showed significant alterations in pathways associated with mitochondrial bioenergetics and synapse organization pathways [52]. Notably, the changes in pathways related to synapses were more significantly affected in the most vulnerable subtypes of dopaminergic neurons in comparison with the more resilient clusters. This study is the first to demonstrate a significant impact of LRRK2 pathogenic mutations on the most susceptible dopaminergic neurons, reinforcing the causal role of this kinase in PD. These findings may also explain the observed reductions in dopamine release in these mutant mice [53–55], correlating synaptic alterations with functional deficits, as further discussed in the following sections.

LRRK2 is expressed across all brain regions, but it is particularly enriched in areas associated with the dopaminergic circuits. These include cortical pyramidal neurons of layer V, medium spiny neurons expressing dopamine receptor D1 and D2, and the thalamus [56]. Its expression in dopaminergic neurons is relatively low; however, it remains detectable across various dopaminergic subtypes [52,57]. We recently found that LRRK2 protein expression is elevated in the subpopulation of dopaminergic neurons that are most vulnerable to PD, specifically in the ventral tier of the SNpc, marked by ALDH1A1, in mice [55]. Complementary human single-nucleus RNA sequencing data show that LRRK2 is enriched in midbrain dopaminergic subtypes marked by SOX6, including the ALDH1A1 + subcluster, which is selectively lost in human PD [55]. This, along with the role of kinases as signal amplifiers, suggests that even modest levels of LRRK2 may be sufficient to contribute functionally to these vulnerable neuronal populations.

## LRRK2 at the synapse: dopamine neurons and beyond

**Figure 2:**
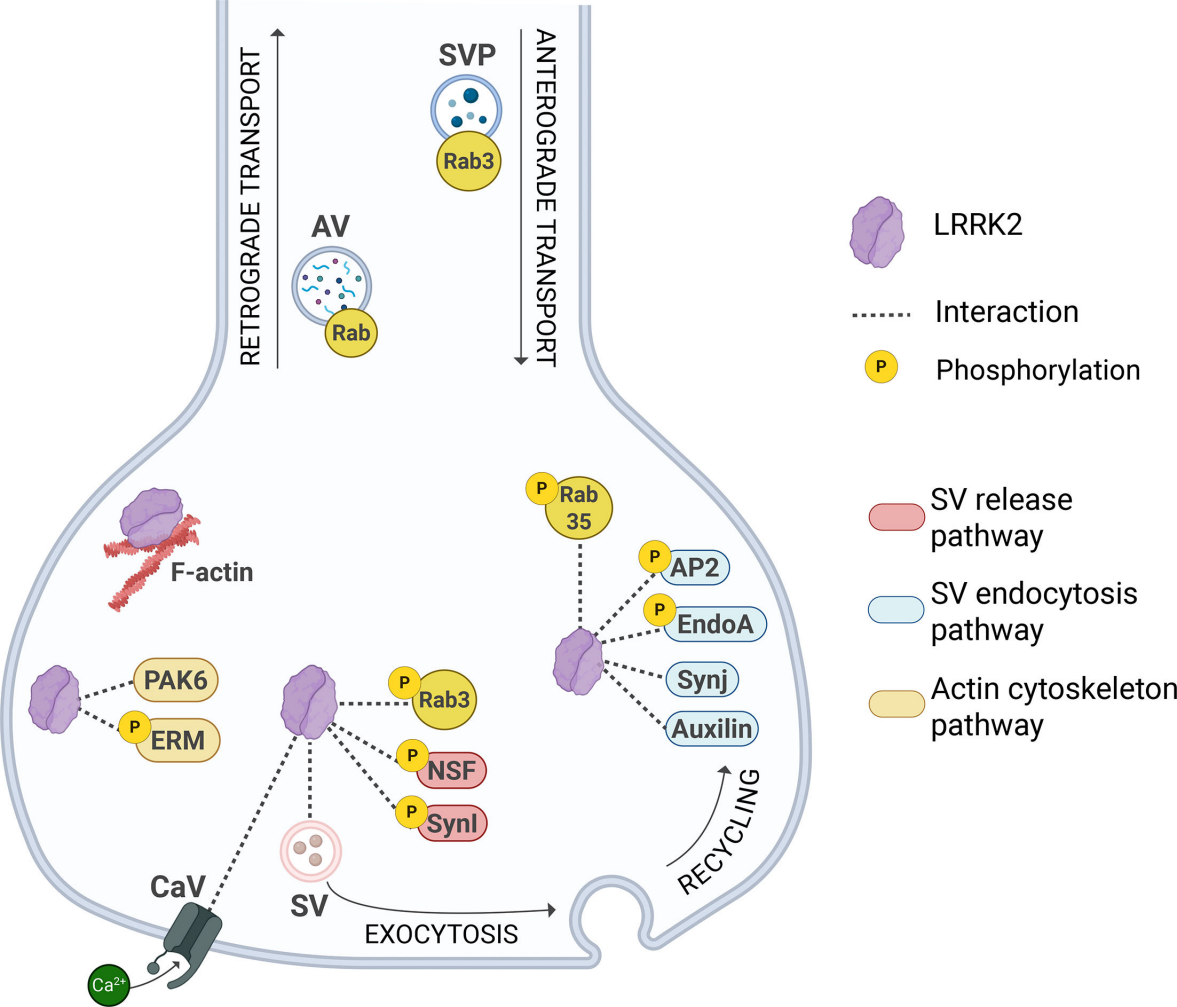
Schematic representation of LRRK2 main interactions at the presynapse. In the actin-cytoskeleton pathway, LRRK2 is reported to bind filamentous actin (F-actin) [59] and to interact with PAK6 and ERM family of proteins [60–63]. In the synaptic vesicle (SV) endocytosis pathway, LRRK2 phosphorylates the AP2 complex subunit M1 [64], endophilin A (EndoA) [65], and Rab35 [66], and interacts with synaptojanin 1 (Synj) [67] and auxilin [68]. Regarding neurotransmitter release, LRRK2 is able to bind SV [69] and to interact with calcium channels (CaV2.1) [70]. Moreover, LRRK2 phosphorylates N-ethylmaleimide-sensitive factor (NSF) [71], synapsin I (SynI) [72], and Rab3 [19]. LRRK2 may also be indirectly involved in disruption of autophagic vesicle (AV) transport and synaptic vesicle precursors (SVP) trafficking through hyperphosphorylation of Rab proteins [73,74].

**Figure 3:**
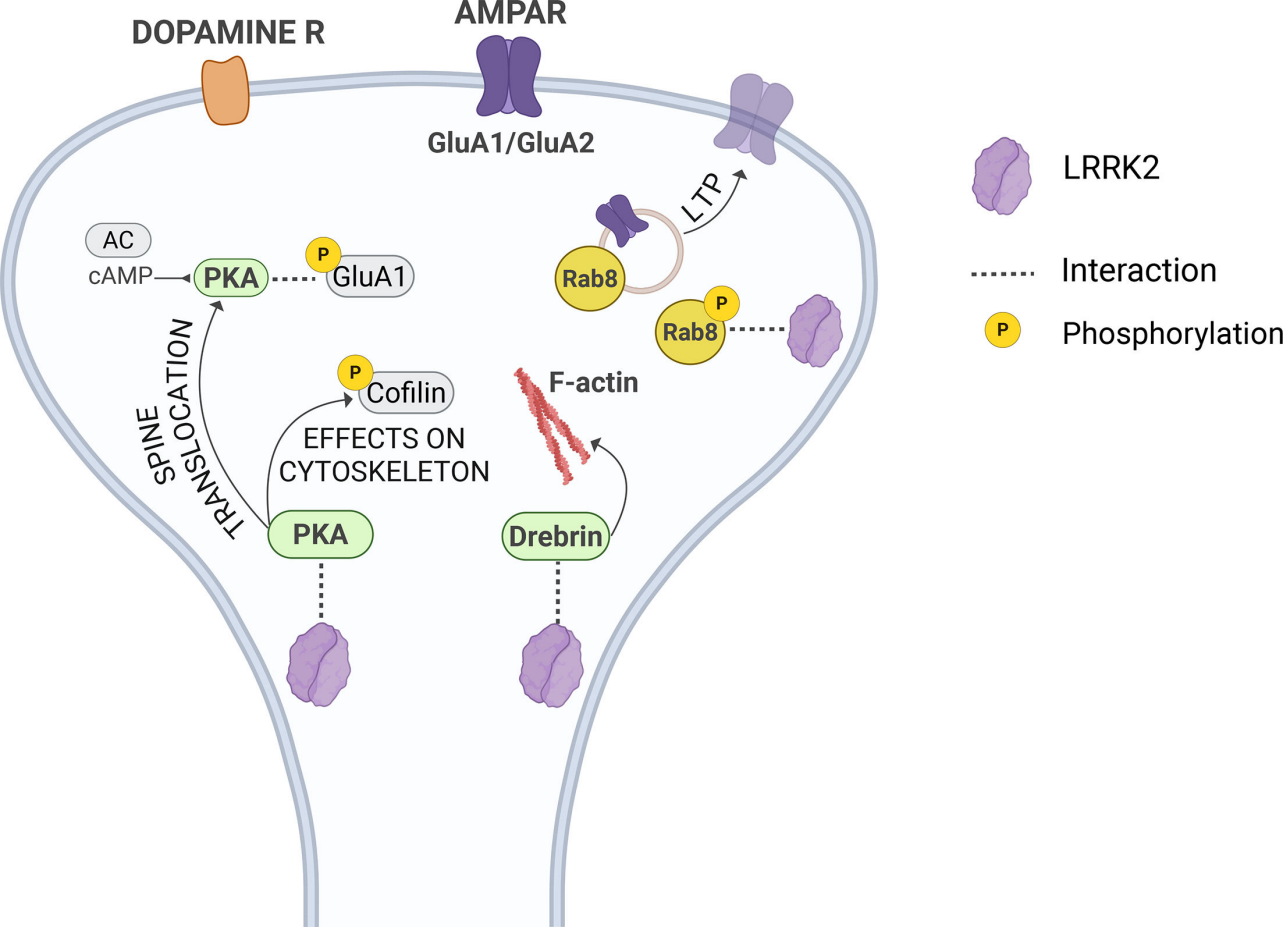
Schematic representation of LRRK2 main interactions at the postsynapse. At the postsynapse, LRRK2 interacts with drebrin [75,76] and with protein kinase A (PKA) regulatory subunit (PKARIIβ), controlling PKA subcellular distribution and consequently GluA1 subunit phosphorylation [77]. Rab8, a validated LRRK2 substrate [19], is involved in α-amino-3-hydroxy-5-methyl-4-isoxazolepropionic acid-type glutamate receptor (AMPAR) trafficking [78]. AC, adenylyl cyclase; F-actin, filamentous actin; LTP, long term potentiation.

While we have only begun to understand the involvement of LRRK2 specifically in the nigrostriatal synapse, a wealth of literature supports its physiological and pathological role at the synaptic compartment. There, LRRK2 influences both pre- and postsynaptic physiology and architecture, primarily through the interactions with key synaptic components [22,58], as illustrated in Figures 2 and 3. Functional studies using knockout and disease-linked mutant LRRK2 cellular and animal models have further demonstrated its regulating role in critical synaptic processes (summarized in Table 1). Here, we summarize and aim to provide a cohesive understanding of the various studies examining how gain-of-function kinase mutations (G2019S), Roc/GTPase mutations (R1441C/G/H), and loss-of-function *Lrrk2* knockouts affect multiple aspects of synaptic physiology.

**Table 1:** LRRK2 pathogenic mutants and knock out (KO) effects on synaptic function

Synaptic feature	LRRK2 condition	Description of effect	Species	Preparation	Region	Reference
Synaptic vesicles (SV) trafficking	KO	Increased SV motility	Mouse	Primary neurons	Cortex	[69]
		Reduced SV endocytosis (aberrant EndophilinA phosphorylation)	Drosophila	Tissue	Neuromuscular junction	[65]
		Decreased α-syn propagation	Rat	Tissue slices (dorsal motor nucleus of the vagus)	Brain	[79]
		Increased neurite outgrowth	Mouse	Primary neurons	Cortex, hippocampus	[77]
	G2019S	Reduced SV endocytosis (impaired endophilin A phosphorylation)	Drosophila	Tissue	Neuromuscular junctions	[65]
		Increased SV dynamics (increased rate of SNARE complex disassembling by aberrant phosphorylation of NSF)	Mouse	Primary neurons	Cortex	[71]
		Increased SV fusion (aberrant synapsin I phosphorylation)	Mouse	Primary neurons	Cortex	[72]
		Hyperphosphorylation of ERM family proteins, retardation of neurite outgrowth	Mouse	Primary neurons	Cortex, hippocampus	[62]
		Hyperphosphorylation of ERM family proteins	Rat	Primary astrocytes	Cortex	[63]
		Disruption of SV endocytosis (endophilin A phosphorylation)	Drosophila	Tissue	Neuromuscular junction	[65]
		Aberrant endocytosis, decreased SV density	Human	iPSC neurons	Dopaminergic neurons	[80]
		Acceleration of SNARE complex disassembly rate	Mouse	Primary neurons	Cortex	[81]
		Increased SV fusion (aberrant synapsin I phosphorylation)	Mouse	Primary neurons	Cortex	[72]
		Increased secretory autophagy	Mouse, human	Neurons	Cortex	[82]
		Decreased binding of Rab3 to RIM proteins due to LRRK2 hyperphosphorylation	Mouse	Synaptosomes	Brain	[52]
		Increased α-syn propagation	Human	SH-SY5Y	Cell	[79]
		Disruption of autophagosomes axonal transport	Mouse	Primary neurons	Cortex	[83]
	R1441C/G/H	RC: reduced SV endocytosis (aberrant auxilin phosphorylation)	Human	iPSC-derived DA neurons	Midbrain	[80]
		RG: reduced SV endocytosis (aberrant auxilin phosphorylation)	Human	iPSC-derived DA neurons	Midbrain	[80]
		RH: disruption of anterograde axonal transport through Rab3 hyperphosphorylation	Human	iPSC neurons (glutamatergic excitatory neurons)	Cortical-like neurons	[74]
		RH: disruption of autophagosomes axonal transport	Human	iPSC-derived neurons	Glutamatergic neurons	[73]
Neurotransmitters release	G2019S	No alterations in striatal evoked dopamine release (12 months)	Mouse	Brain slices (FSCV)	Striatum	[84]
		Increased presynaptic glutamate transmission in young mice (1–3 months)	Mouse	Brain slices (whole-cell patch clamp recording)	Striatum	[84]
		Reduced evoked dopamine release	Mouse	FSCV in striatal tissues	Striatum	[84]
		Functional alterations in neuronal networks	Human	iPSC neurons	Dopaminergic neurons	[53]
		Significantly reduced evoked dopamine release	Mouse	Fiber photometry (dopaminergic neurons)	Dorsal striatum	[85]
		Reduced evoked dopamine release	Mouse	FSCV in striatal tissues	Striatum	[52]
	R1441C/G/H	RC: reduced AMPH-induced dopamine release	Mouse	Adrenal chromaffin cells (amperometric recording)	Adrenal medulla	[53]
		RC: no alterations in dopamine levels (3, 12, and 22 months)	Mouse	Tissue (HPLC)	Striatum	[86]
		RC: increased GluA1 phosphorylation	Mouse	Tissue (WB)	Forebrain	[86]
Postsynaptic receptor trafficking	KO	Reduced D1 receptor internalization	Human	SH-SY5Y	Cell	[87]
	GS	Reduced D2 receptor turnover	Human	SH-SY5Y	Cell	[88]
		Increased GluA1 receptor accumulation in the dSPNs synapse	Mouse	Primary neurons	Striatum-cortex	[88]
		Decreased levels of calcium-permeable AMPA receptors in striatal synapses	Mouse	Brain slices (whole-cell patch-clamp)	Striatum	[89]
		Impaired D2 receptor functions	Mouse	Brain slices (electrophysiological recordings)	Striatum	[90]
	R1441C/G/H	RC: increased GluA1 levels in dendritic spines of dSPNs	Mouse	Synaptosomes	Striatum	[86]
Dendritic spines	KO	Reduced number of mature dendritic spines (p15 pups)	Mouse	Brain slices (Golgi)	Striatum	[91]
		Increased PKARIIβ levels in dendritic spines	Mouse	Tissue (co-IP)	Striatum	[87]
		Increased cofilin phosphorylation	Mouse	Brain homogenate	Striatum	[87]
		Smaller dendritic spines (1 month)	Mouse	Brain slices (Golgi)	Striatum	[75]
	GS	No alterations in dendritic spine density and morphology (1 month)	Mouse	Brain slices (viral injection)	Striatum	[92]
		No alterations in dendritic spine density (1–3 months and 12 months)	Mouse	Brain slices (Golgi)	Striatum	[84]
		Increased drebrin phosphorylation	Human/mouse	SH-SY5Y/striatum brain homogenate	Striatum	[75]
		Larger dendritic spines (increased dendritic spine head width)	Mouse	Brain slices	Striatum	[90]
	R1441C/G/H	RC: decreased spines area	Mouse	Primary neurons	Hippocampus	[87]
		RC: increased PKARIIβ signal in dendritic spines	Mouse	Primary neurons	Hippocampus	[87]
		RC: no alterations in dendritic spine density and morphology (1 month)	Mouse	Brain slices (viral injection)	Striatum	[91]
		RC: increased PKA signaling in PSD fractions of striatal extracts	Mouse	Synaptosomes	Striatum	[91]
Post-synaptic currents	KO	Reduced mEPSCs frequency	Mouse	Brain slices (whole-cell voltage clamp recording)	Striatum	[87]
		Reduced mEPSCs frequency in dSPNs and iSPNs	Mouse	Brain slices (whole-cell voltage clamp recording)	Striatum	[91]
		No alterations in glutamatergic eEPSC amplitudes (3–6 months)	Mouse	Brain slices (whole-cell patch clamp recording)	Striatum	[93]
		No alterations in glutamatergic sEPSC amplitudes and frequencies (3–6 months)	Mouse	Brain slices (whole-cell patch clamp recording)	Striatum	[93]
	GS	Increased sEPSC frequency (p21 mice)	Mouse	Brain slices (whole-cell patch clamp recording)	Striatum	[90]
		No alterations in glutamatergic sEPSCs and in eEPSCs (striatal SPNs)	Mouse	Brain slices (whole-cell patch clamp recording)	Striatum	[54]
		Increased glutamatergic sEPSC frequency (1–3 months)	Mouse	Brain slices (whole-cell patch clamp recording)	Striatum	[84]
		No alterations in glutamatergic sEPSC frequency (12 months)	Mouse	Brain slices (whole-cell patch clamp recording)	Striatum	[84]
		Deficits in corticostriatal connectivity	Mouse	Behavioral tests	Brain	[94]
		Decreased cholinergic innervation	Mouse	Tissue slice (immunolabeling)	Cortex, striatum	[94]
		Age-dependent increased D1 response	Mouse	Behavioral tests	Striatum	[95]
		No alterations in intrinsic membrane properties and action potential properties	Mouse	Brain slices (cell-attached and whole-cell patch clamp)	SNpr	[96]
	R1441C/G/H	RC: reduced mEPSCs frequency in dSPNs and iSPNs	Mouse	Brain slices (whole-cell voltage clamp)	Striatum	[91]

dSPNs, direct spiny projection neurons. iSPNs, indirect spiny projection neurons. eEPSC, evoked excitatory postsynaptic current. mEPSC, miniature excitatory postsynaptic current. SV, synaptic vesicles. SNARE, soluble NSF attachment protein receptor. NSF, N-ethylmaleimide–sensitive factor. α-syn, alpha synuclein. PSD, postsynaptic density. sEPSC, spontaneous excitatory postsynaptic current.

### LRRK2 at the presynapse: a link between cytoskeleton and vesicle trafficking

Multiple studies have demonstrated that LRRK2 participates in the organization of the actin-based cytoskeleton, which is crucial for neuronal development and activity-dependent shaping of pre- and postsynaptic components. An early interactome study in NIH3T3 cells showed that LRRK2 associates with various actin isoforms and actin-associated proteins. Specifically, LRRK2 binds filamentous actin (F-actin) and modulates its assembly *in vitro* [59]. Moreover, LRRK2 is necessary to promote PAK6-dependent neurite outgrowth in the mouse striatum through the LIMK1–cofilin–actin pathway [60,61]. It has also been shown that LRRK2 phosphorylates members of the ERM protein family (ezrin, radixin, and moesin) in both cultured neurons and astrocytes. These proteins are essential for cross-linking F-actin filaments to the plasma membrane and exhibit hyperphosphorylation in G2019S LRRK2 primary neurons and astrocytes, leading to defects in cell morphology [62,63].

In 2011, Piccoli et al. demonstrated in cultured neurons that LRRK2 can bind to SV via its WD40 domain, supporting its role as a scaffold protein. This positions LRRK2 as a molecular bridge between vesicle trafficking and the cytoskeleton at the synapse [69]. This hypothesis was further reinforced by subsequent findings showing that LRRK2 phosphorylates SV-associated proteins, as discussed below.

Clathrin-mediated endocytosis at the synapse is an essential process for the regeneration of SV pools, strictly controlled by phosphoregulated proteins, including synaptojanin 1 and dynamin 1 [97]. This tightly regulated mechanism is initiated by clathrin recruitment through the adaptor protein AP2, followed by membrane invagination facilitated by endophilin A and synapojanin 1, and terminates with auxilin that removes clathrin. Notably, LRRK2 is reported to interact with all of these proteins, highlighting its role in finely tuning SV endocytosis [75]. Accordingly, LRRK2-mediated phosphorylation of the AP2 complex subunit M1 is essential for initiating clathrin coating of budding endocytic vesicles, as demonstrated in cultured neurons and in animal models [64]. Furthermore, LRRK2 also phosphorylates endophilin A, and studies in *Drosophila* have shown that both reduced and increased LRRK2 activity have an impact on efficient vesicle formation [65].

Auxilin is a co-chaperone for heat shock cognate 70 that plays a critical role at the synapse by mediating the uncoating of clathrin-coated vesicles [68]. It has also been identified as an LRRK2 interactor. A study, using human induced pluripotent stem cell (iPSC) models of dopamine neurons with mutant LRRK2 (R1441C or G2019S), revealed abnormal SV endocytosis, with decreased SV density [80]. These defects led to increased levels of oxidized dopamine and α-syn in dopaminergic neurons. Both of these toxic effects were linked to increased kinase activity-dependent LRRK2 phosphorylation of auxilin [80].

Additionally, phosphorylation by LRRK2 is required for the macroautophagic role of endophilin A, which is at least in part independent from its role in endocytosis [98]. LRRK2’s involvement in synaptic autophagy was further validated by the discovery of the interaction with synaptojanin 1, which promotes SV recycling. This interaction, first identified through an interactome study in *Drosophila*, was later confirmed with an *in vitro* radioactive kinase assay of human LRRK2 and human SYNJ [67]. Interestingly, studies in both iPSCs and rodent cellular models have reported a disruption of maturation and axonal transport of autophagosomes due to LRRK2 mutations (G2019S and R1441H) [73,83], directly linking pathological LRRK2 activity to defects in neuronal autophagy. A recent study reported upregulation of secretory autophagy in both murine and human neurons expressing G2019S LRRK2 [82], suggesting that this increased secretion contributes to the maintenance of cellular homeostasis, delaying neurodegenerative disease progression. However, the precise mechanistic role of LRRK2 activity bridging neuronal autophagy and synaptic function remains incompletely understood. Of interest, a preprint study reported PAK6, a kinase enriched in synaptic compartments, as a novel regulator of neuronal autophagy [71]. The study shows how PAK6 activity reduces the toxic accumulation of α-syn, both in *Drosophila* and in dopaminergic neurons of a mouse model of LRRK2-PD. Considering the established interaction between LRRK2 and PAK6 [60], this pathway may represent a potential missing link in the mechanism by which LRRK2 disrupts neuronal degradative pathways.

### Effects of LRRK2 kinase activity on neurotransmitter release

The release of neurotransmitters from SV is a core aspect of chemical neurotransmission, and growing evidence implicates LRRK2 in regulating this process. LRRK2 has been shown to interact with N-ethylmaleimide-sensitive factor (NSF), an ATPase critical for SNARE complex disassembly during SV exocytosis, both in cell lines (HEK cells and cortical neurons) [71], and *in vivo* murine models [81]. LRRK2-mediated NSF phosphorylation at T645 accelerates the SNARE complex disassembly [99], which can affect both neurotransmitter release and SV endocytosis. Moreover, mice expressing human LRRK2 G2019S exhibit age-related motor and cognitive deficits, along with the formation of NSF aggregates [81]*.* Additionally, LRRK2 interacts with synapsin I, a key protein for SV clustering and their tethering to the actin cytoskeleton [72]. Phosphorylation by LRRK2 weakens this interaction, disrupting SV dynamics. In this study, it was also suggested that LRRK2 may mediate glutamate release in a kinase-dependent and synapsin I-dependent manner, as glutamate release was reduced in both cortical synaptosomes with LRRK2 inhibition and in *SynI* knockout mice, while inhibition of LRRK2 in *SynI* knockout mice had virtually no effects [72].

Along these lines, Volta and collaborators demonstrated age-dependent alterations in glutamate transmission in brain slices of young G2019S knock-in mice. They observed significant increases in event frequency but no changes in synapse numbers. This effect is concomitant with changes in dopamine release regulation in young animals [84], consistent with findings from another study reporting reduced dopamine release in both R1441C and G2019S mice [52]. Moreover, a study conducted in complex functional networks formed by human iPSC-derived dopaminergic neurons from patients harboring the G2019S LRRK2 mutation further supports the detrimental effect of mutant LRRK2 on dopaminergic neurotransmission. Specifically, mutant dopaminergic neurons displayed reduced connectivity, suggesting an overall network dysfunction, a lack of functional microcircuits, and a tendency toward excessive network synchronicity [85].

Lastly, LRRK2 also influences synaptic transmission through interaction with calcium channels, specifically via CaV2.1, which facilitates the entry of calcium ions upon action potential-induced depolarization. In HEK293T cells, LRRK2 was shown to increase whole-cell calcium current density through a kinase-dependent interaction with the β3 subunit of CaV channels [70]. Whether LRRK2 regulates activity-dependent calcium entry in dopaminergic neurons in a similar fashion remains to be determined.

### Rab GTPases and their role at the synapse

Rab GTPases are key regulators of vesicular trafficking, with some members also present at the synaptic compartment [100]. This large family consists of approximately 60–70 members, with around 10 GTPases identified by several independent laboratories as physiological LRRK2 substrates [19,66]. Among those, Rab3a is an essential regulator of the SV cycle, as it binds to SV and interacts with several SV-associated proteins [101–103]. Rab3a is involved in the docking and fusion of SV with the presynaptic membrane and contributes to the anterograde axonal transport of synaptic vesicle precursors (SVP) [104]. Importantly, a study in iPSC-derived human neurons has functionally connected LRRK2 with Rab3a, showing that Rab3a hyperphosphorylation due to pathogenic LRRK2 impairs the proper transport of SVP from the soma to the synaptic site [74]. Recently, Chen and collaborators used affinity purification coupled with mass spectrometry in mouse brain homogenate to demonstrate that the phosphorylation of Rab3a by LRRK2 disrupts its interaction with its effectors RIM1 and RIM2. These proteins are essential scaffolds in the presynaptic active zone, which is necessary for neurotransmitter release [55]. These findings support the idea that excessive LRRK2 activity, due to PD-linked mutations, alters the presynaptic architecture by disrupting Rab3a interactions with its effectors.

Another LRRK2 substrate, Rab35, has been identified as a putative regulator of post-endosomal SV trafficking, as demonstrated by functional screens performed in *Drosophila* [105]. Rab35 also plays an important role in the regeneration of SV, as it facilitates their degradation and turnover. Specifically, Rab35 recruits the Endosomal Sorting Complex Required for Transport (ESCRT)-O protein Hsr to SV pools, promoting the degradation of synaptic vesicles protein 2 and synaptobrevin-2, as demonstrated in cultured rat hippocampal neurons [106]. Importantly, a study demonstrated that LRRK2 plays a crucial role in α-syn propagation, using *in vivo* rodent models, and that this is mediated by Rab35 phosphorylation [79]. The authors suggested that excessive Rab35 phosphorylation may cause dysregulation of α-syn clearance, particularly at the interface between lysosomal degradation and endosomal recycling [79]. It is possible to hypothesize a similar scenario at the synapse, where excessive Rab35 phosphorylation driven by hyperactive LRRK2 leads to disrupted vesicle turnover.

Additionally, evidence from *C. elegans* suggests that Rab10 may contribute to synaptic function by regulating neurotransmitter release from dense core vesicles (DCV) [107]. In cortical neurons, Rab10 is known to regulate polarized membrane trafficking during axon development [108], and it is involved in clathrin-mediated endocytosis and recycling pathways [109]. Rab10 is one of the best-established substrates of LRRK2 [19,110]; thus, it would be interesting to explore whether increased phosphorylation of synaptically localized Rab10 affects DCV, which, as discussed below, play a crucial role in dopamine storage and release.

These findings highlight the crucial role of LRRK2 in managing the trafficking, release, and recycling of SV. Efficient turnover of proteins at the synapse is particularly important due to its distance from the cell body and the need for local, autonomous regulation. As a result, any disruptions in synaptic protein balance, along with impaired neurotransmission, are likely to be key early events that contribute to neurodegeneration.

### LRRK2 effects on postsynaptic signaling

Besides orchestrating important functions at the presynaptic site, LRRK2 has been shown to influence the postsynaptic compartment of excitatory synapses in the striatum, affecting synaptic development, transmission, and plasticity [75,76,92,111]. Disease-linked G2019S and R1441C mutations alter both glutamatergic and dopaminergic signaling [84,86,93]. However, the precise molecular mechanisms by which LRRK2 regulates postsynaptic signaling pathways and synaptic remodeling remain unclear.

Due to the high expression of LRRK2 in medium spiny neurons, research focused on understanding the function of LRRK2 in these neurons is highly relevant, as they are part of the nigrostriatal and corticostriatal pathways, both affected in PD. This is of particular significance when considering the dying-back hypothesis of nigrostriatal axons [9] and the corticostriatal-based cognitive impairments [112] recognized as early non-motor PD symptoms. Accordingly, several studies have reported that pathological LRRK2 kinase activity affects synaptic transmission in these circuits. Mice carrying the G2019S mutation exhibit deficits in cognitive tasks that rely on frontal-striatal connectivity and cholinergic modulation, with improvement in their performance following administration of an acetylcholinesterase inhibitor. Additionally, in the G2019S mice, cortical cholinergic innervation was significantly sparser compared with wildtype animals [94]. These results suggest a contribution of mutated LRRK2 to early cognitive dysfunction through cholinergic signaling. Furthermore, corticostriatal synapses of G2019S mice exhibit abnormal synaptic plasticity, failing to express long-term potentiation (LTP) due to failed accumulation of functional calcium-permeable α-amino-3-hydroxy-5-methyl-4-isoxazolepropionic acid-type glutamate receptors (AMPARs) [90]. Given the fundamental role of AMPARs in plasticity events in glutamatergic synapses, this points to a potential early adaptive coping mechanism involving LRRK2 [90]. Supporting this, a recent study showed both biochemical and functional alteration of AMPAR subunit stoichiometry in G2019S mice, with spiny projection neurons GluA1 incorporation over GluA2. Because GluA1-containing AMPARs are resistant to internalization, they accumulate excessively within and outside synapses, negatively impacting receptor trafficking and synapse strengthening [89]. Collectively, these findings indicate that the defective plasticity observed in G2019S mutants is, at least in part, driven by altered AMPAR expression and trafficking dynamics.

LRRK2 may also play a direct role in dopaminergic signaling by interacting with dopamine receptors D1 and D2. Rassu et al. [88] demonstrated that LRRK2 G2019S mutation impairs D1 internalization, leading to alterations in signal transduction [88]. Moreover, this mutation causes defects in D2 turnover, affecting the trafficking from the Golgi complex to the cell membrane [88]. This finding was confirmed by a recent study, in which G2019S mutation was found to be associated with an age-dependent increase in D1 responses, likely due to failed internalization of the receptor [95]. A recent study by our group provided additional evidence of LRRK2’s involvement in dopamine receptor pathways. In particular, the authors demonstrated that the inhibition of LRRK2 kinase activity rescues the PD-like side effects of haloperidol, an antagonist of D2, and promotes recovery of structural and functional haloperidol-induced changes. On the contrary, LRRK2 hyperactivity mimics the molecular and biochemical effects of haloperidol on striatal intracellular signaling. Overall, this points to an inhibitory effect of LRRK2 on D2 signaling, potentially resulting in decreased D2-dependent inhibition of cAMP-dependent [protein kinase A (PKA)] and independent (β-arrestin 2) cascades [113].

LRRK2 was also shown to alter striatal circuit architecture by decreasing the number of mature spines, particularly during the developmental stage [87]. An examination of 1-month-old *Lrkk2* knockout striatum revealed a significantly shorter postsynaptic density (PSD) compared with wildtype, which is a direct indicator of the density of postsynaptic receptors and signaling proteins [75]. Moreover, increased kinase activity due to the G2019S mutation leads to abnormal morphology and gain-of-function activity of dorsal striatal medium spiny neurons during postnatal development, a stage that is pivotal for the permanent formation of circuit structure and function [111]. These data point to a possible involvement of LRRK2 in postsynaptic spine development.

There is still no cohesive and clear synthesis of how pathogenic LRRK2 affects postsynaptic currents in adult mice. One study reported that the glutamatergic alterations caused by the G2019S mutation are not maintained in adult mice [84], while a more recent publication on spiny projection neurons has shown a reduction in miniature excitatory postsynaptic currents (mEPSCs) frequency in R1441C and, to a lesser extent, G2019S acute brain slices from adult mice [91]. Another study has investigated characteristics of glutamatergic synaptic transmission in basal ganglia output neurons, the substantia nigra pars reticulata (SNpr), finding an overall maintenance of basic neurophysiological properties in G2019S mice [96]. Variability in experimental approaches and conditions across studies could explain this discrepancy, underscoring the need for further investigation on this topic.

### Molecular basis of LRRK2-mediated postsynaptic effects

Multiple studies have explored the molecular mechanisms involved in LRRK2-mediated changes at the postsynaptic level, highlighting the pathways and processes affected by this kinase in the postsynaptic compartment.

Parisiadou et al. [87] suggested that LRRK2 acts as a negative modulator of PKA activity in spiny projection neurons during synaptogenesis and in response to dopamine receptor activation after the postnatal developmental period. In more detail, LRRK2 interacts with PKARIIβ regulatory subunit, controlling its subcellular distribution. This interaction is significantly diminished by the LRRK2 R1441C mutation. As a result, there is an increased PKA translocation to dendritic spines, which brings it closer to the upstream components of cAMP-mediated signaling transduction pathways, such as dopamine receptors and adenylate cyclase, as well as the downstream substrates, such as GluA1 [77]. Specifically, PKA-mediated phosphorylation of GluA1 enhances its incorporation into the synaptic membrane, which aligns with observations by Gupta et al. (2004) [89]. Moreover, Parisiadou and colleagues found that *Lrrk2* knockout mice show an aberrant phosphorylation of cofilin, an actin-binding protein, which appears to impair actin dynamics and spine formation [77]. Although LRRK2 does not directly interact with cofilin, it acts on PKA, which in turn activates upstream kinases such as LIMK1 that phosphorylates cofilin [77].

Although multiple lines of evidence support a role for LRRK2 in dopaminergic signaling, particularly through its interaction with PKA, the opposite effects of the direct and indirect striatal pathways (D1 and D2 receptors, respectively) on PKA activity following dopamine receptor activation [114] raise questions about whether LRRK2 influences both pathways equally or preferentially. Future research should investigate whether LRRK2 mutations exert cell type-specific effects to gain a more nuanced understanding of the underlying molecular mechanisms.

Rab8a, a validated LRRK2 substrate, has been identified as a key player in the cellular mechanism of AMPAR trafficking at the synapse. Rab8a was shown to localize in close proximity to the synaptic membrane, including the PSD, and electrophysiological studies indicated Rab8a as necessary for the local AMPAR delivery into the spine surface during LTP and for the constitutive receptor cycling [78]. This has been confirmed by a study that isolated AMPAR-containing vesicles from mouse brain and identified Rab8 as one of the major hits in mass spectrometry [115]. Whether Rab8a phosphorylation by pathogenic LRRK2 has any effects on the trafficking of these receptors is currently unknown, and further research is needed to elucidate the mechanisms underlying the effects of LRRK2 on AMPAR delivery.

Additional evidence of a role for LRRK2 in the postsynaptic compartment has recently emerged from an affinity purification study in SH-SY5Y cells stably expressing LRRK2. In this study, one-third of LRRK2 interactors were annotated in the SynGO database [116]. Among these interactions, 66% were associated with the postsynaptic compartment, while 33% corresponded with presynaptic proteins. Notably, the most significantly enriched functional categories included pre- and postsynaptic ribosomes, as well as postsynaptic actin cytoskeleton [75]. Building on these findings, a PPI network centered on LRRK2 identified a cluster of 41 genes highly enriched in actin cytoskeleton and synaptic categories. Among them, *DBN1*/drebrin, an actin-binding protein crucial for co-ordinating dendritic spine morphology, emerged as a key element in the cluster. This protein was found to be hyperphosphorylated in hyperactive LRRK2 G2019S knock-in mice [75,116].

Together, these findings strengthen the evidence for LRRK2 localization at the synapse and support a model in which LRRK2 functions as a scaffold protein that modulates postsynaptic signaling by remodeling the actin cytoskeleton, possibly influencing receptor trafficking and activation.

### A role for LRRK2 at the dopaminergic synapse?

Recent evidence has refined our understanding of dopamine transmission. Only about 20–30% of dopamine varicosities harbor active zone-like release sites and engage in action potential-driven exocytosis, while the majority remain silent under depolarization [117]. These silent sites may still be activated by local cholinergic neurotransmission, which is itself linked to LRRK2 signaling and altered in LRRK2 patients and preclinical models [118–120]. This cholinergic activity can trigger dopamine release independently of somatic firing, linking non-active varicosities to striatal circuit activity in ways that go beyond canonical action potential propagation [121]. Overall, it is accepted that additional work is required to uncover the secretory biology of dopamine neurons.

What we know for dopaminergic synapses is that their active zone machinery has different protein requirements than fast glutamatergic synapses [121,122]. Indeed, dopamine release is strongly dependent on the scaffold RIM1/2, whereas ELKS (aka ERC1/2) plays a comparatively minor role [123]. This unique dependence on RIM makes dopamine synapses particularly vulnerable to molecular disruptions that weaken RIM function. As previously mentioned, our recent work shows that LRRK2 phosphorylates Rab3a at T86, reducing its interaction with both RIM1 and ERC2 [55]. Since RIM functions are necessary for dopamine synapses that rely on rapid action potential-triggered release, the disruption of Rab3a-RIM1 interaction by LRRK2 could have particularly harmful effects on dopaminergic neurons. This impairment may affect the sparse release-competent varicosities that sustain phasic dopamine signaling.

In addition, a subset of dopamine varicosities establishes *bona fide* gamma-aminobutyric acid (GABA)-ergic synapses, with presynaptic neurexin and ERC2 aligned with postsynaptic neuroligin-2 clusters [124]. These structures support GABA co-release from dopamine axons, further diversifying their output. By destabilizing Rab3a-RIM interactions, mutant LRRK2 could therefore disrupt not only dopamine release but also inhibitory co-transmission, biasing the balance of striatal signaling.

Dopaminergic synapses are characterized by the coexistence of small clear SV and large dense-core vesicles (LDCV) [125,126]. SV and LDCV have distinct origins and life cycles. While SVs are generated from precursor vesicles transported from the soma but are largely sustained by local recycling at presynaptic terminals [127,128], LDCVs are formed at the trans-Golgi network and then transported along axons and dendrites to release sites with no recycling mechanisms [129]. Given that LRRK2 regulates both cytoskeletal dynamics [61,83,130] and trans-Golgi network function, e.g. upon casein kinase 1α phosphorylation and interaction with the GARP complex [131,132], one hypothesis is that pathogenic LRRK2-mediated perturbations of trans-Golgi processing and/or axonal transport could selectively impair LDCV biogenesis and delivery.

In conclusion, we can hypothesize that LRRK2 mutations destabilize dopamine-containing vesicles by interfering with their trafficking and/or exocytosis. Future studies will be essential to determine whether LRRK2 exerts a preferential impact on LDCV compared with small SV, an issue that could provide important insights into the selective vulnerability of dopaminergic neurons.

## Neurotrophins, Parkinson’s disease, and LRRK2

Neurotrophic factors such as glial-derived neurotrophic factor (GDNF) and BDNF are crucial for the survival and plasticity of dopaminergic neurons and medium spiny neurons during development and adulthood. They promote neuronal health by activating pro-survival signaling pathways, regulating dopamine synthesis and release, and protecting neurons from stress and degeneration [133,134]. Exposure to GDNF increases the quantal size of dopamine release from axonal varicosities [135], and *in vivo* studies in rodents and primates have shown that GDNF enhances evoked striatal dopamine release, elevates tyrosine hydroxylase phosphorylation, boosts dopamine synthesis, and increases the number of dopamine neurons and their terminals in the striatum [125,136]. GDNF signaling through its canonical Ret receptor also regulates DAT trafficking, thereby influencing dopamine reuptake and homeostasis [137].

Emerging evidence indicates that LRRK2 mutations impair the formation and function of primary cilia in the striatum, particularly in cholinergic interneurons and parvalbumin neurons [138]. Since Hedgehog (Shh) signaling, which relies on cilia, regulates GDNF expression, LRRK2 mutations may disrupt Shh signaling, leading to reduced GDNF production in these neurons [138,139]. This deficiency could compromise the GDNF-mediated neuroprotective support of dopaminergic neurons, potentially contributing to LRRK2-associated PD pathogenesis. Although mice carrying LRRK2 mutations do not exhibit overt signs of neurodegeneration, they display striatal synaptic impairments, which may be at least partly due to lower GDNF levels. Notably, these deficits, as well as GDNF levels, are rescued by *in vivo* inhibition of LRRK2 kinase [140].

BDNF, acting primarily through TrkB and p75 receptors, likewise stimulates dopamine turnover and release *in vitro* and *in vivo* [141]. Partial *Bdnf* deletion in mice causes age-dependent reductions in VMAT2 and DAT activity, evoked dopamine release, and motor performance, all of which can be partially rescued by exogenous BDNF [142]. BDNF concentration is significantly lower in the SNpc region of PD patients [143], and postmortem tissue analysis has revealed that BDNF mRNA expression is also reduced. This reduction is only partially due to a loss of BDNF-expressing neurons, as surviving dopaminergic neurons also express lower BDNF mRNA levels. Moreover, loss of BDNF seems to precede a reduction in the size of neuronal cell bodies, suggesting that a lowering of BDNF levels might increase vulnerability and subsequently trigger neuronal death [144]. Interestingly, a study in PD patients showed that loss in striatal DAT binding, measured with single photon emission computed tomography (SPECT), correlates with decreased serum BDNF levels [145]. This supports the connection between lower concentration of BDNF and loss of dopaminergic neurons.

BDNF/TrkB signaling has been linked to α-syn pathology. In particular, inclusions seeded by α-syn preformed fibrils (PFFs) are associated with a decrease in BDNF mRNA in cultured neurons, while α-syn overexpression causes BDNF down-regulation [146]. Additionally, α-syn seems to impair retrograde endocytic transport of BDNF, leading to neuronal atrophy [147]. However, *Snca* knockout does not affect the neurotrophic effects of BDNF, indicating that α-syn is not required for BDNF/TrkB physiological functions [147]. The detrimental effects of α-syn on BDNF may stem from its pathological interaction with the TrkB receptor, as α-syn has been shown to selectively bind the kinase domain of TrkB. This interaction disrupts TrkB signaling, internalization, axonal trafficking, and protein stability by inducing its ubiquitination, ultimately leading to loss of dopaminergic neurons [148]. Furthermore, TrkB haploinsufficiency causes SNpc neuronal loss in aged mice, together with an accumulation of α-syn [149].

What about LRRK2? A study conducted on a Taiwanese population found a significant interaction effect between pesticide exposure and genetic variants of *BDNF*, particularly the V66M polymorphism, and *LRRK2* (S1647T polymorphism) [150]. In a subsequent case-control study, researchers analyzed the combined effect of *BDNF* p.V66M and *LRRK2* p.G2385R, which is a common risk factor for PD in the Chinese population [151]. The results revealed a strong additive effect between the two genetic variants, with the BDNF variant nearly doubling the risk for PD conferred by the LRRK2 variant alone in late-onset PD patients [152]. These observations support the hypothesis that an imbalance in BDNF/LRRK2 signaling could lead to oxidative-driven dysfunction in PD pathogenesis.

Supporting these genetic findings, our recent collaborative study has provided compelling evidence that BDNF increases LRRK2 activity and promotes its recruitment to the actin cytoskeleton [75,76]. *Lrrk2* knockout neurons show reduced BDNF-induced activation of downstream AKT and ERK1/2, suggesting that LRRK2 acts downstream of BDNF-TrkB and upstream of MAPK/ERK and PI3K/AKT pathways. BDNF-activated LRRK2 promotes Rab phosphorylation and forms complexes that are enriched in actin-regulating and synaptic proteins, including drebrin [75]. In parallel, the striatal phosphoproteome associated with G2019S LRRK2 was found to be significantly enriched in synaptic proteins, including elevated levels of phosphorylated drebrin. LRRK2 was also shown to influence dendritic spine morphology in a BDNF-dependent manner, in line with previous studies [77]. While *Lrrk2* knockout delayed dendritic development in early stages, this phenotype was later rescued, likely due to compensatory effects from its homologous kinase LRRK1 [153–155]. While the potential detrimental effects of pathogenic LRRK2 on BDNF-induced pro-survival signaling in dopaminergic neurons have not yet been mechanistically defined, we speculate that reducing LRRK2 activity could help restore BDNF signaling. Future studies should investigate this hypothesis to clarify the underlying mechanisms and therapeutic potential.

While a substantial body of evidence links dysfunctional BDNF signaling to PD progression, the therapeutic potential of this pathway is still unclear [156]. In animal models, BDNF treatment improved dopaminergic neuron survival when administered before PD onset or induced a partial recovery of dopaminergic transmission [156]. However, the main limitation for the therapeutic application of BDNF is the difficulty in delivering growth factors to the central nervous system (CNS). Indeed, BDNF has a short half-life, low bioavailability, and poor permeability through the blood-brain barrier. Consequently, to effectively reach brain neurons, BDNF must be administered directly into the CNS, which hampers its therapeutic application, particularly for a chronic regime [156,157]. One alternative approach to augmenting brain BDNF could be through gene therapy. However, previous attempts have been unsuccessful. For example, a clinical trial for Alzheimer’s disease where nerve-growth factor (NGF) was delivered via adeno-associated viral vectors (AAV2–NGF) failed due to the limited spread of AAV2-NGF to target cholinergic neurons [158].

## Conclusions

In summary, we have critically examined the increasing evidence linking aberrant LRRK2 activity to early synaptic dysfunction, highlighting its impact on vesicle trafficking, receptor dynamics, and actin remodeling. Recent studies further revealed that these alterations are especially pronounced within the most vulnerable neuronal populations of the SNpc, establishing a direct link between pathological LRRK2 activity and loss of dopaminergic synapses. Thus, dysfunctional LRRK2-mediated synaptic disturbance may trigger a cascade of events that leads to dopaminergic neurodegeneration through multiple, non-mutually exclusive mechanisms. Importantly, pathogenic LRRK2 also affects other connections, including cholinergic and glutamatergic synapses, in line with their documented involvement in PD. Moreover, the synaptic interactions of LRRK2 with the actin cytoskeleton are modulated by BDNF-TrkB signaling pathways, suggesting that aberrant LRRK2 activity disrupts BDNF-induced synaptic plasticity and neuronal survival. LRRK2 activity also shapes dopaminergic neuron survival indirectly through cilia-dependent GDNF release by striatal neuronal populations, thereby supporting dopaminergic viability.

In conclusion, LRRK2 clearly represents a crucial piece of the puzzle in understanding how vulnerable dopaminergic neurons degenerate in PD. Correcting its pathological activity to physiological levels could not only restore synaptic function but also neurotrophin signaling, offering a promising therapeutic avenue that circumvents the challenges associated with directly delivering growth factors to the brain.

## Open Access

This article has been published open access under our Subscribe to Open programme, made possible through the support of our subscribing institutions, learn more here: https://portlandpress.com/pages/open_access_options_and_prices#conditional

## Abbreviations

AAV: adeno-associated viral vector
α-syn: alpha-synuclein
AMPAR: α-amino-3-hydroxy-5-methyl-4-isoxazolepropionic acid-type glutamate receptors
BDNF: brain-derived neurotrophic factor
CNS: central nervous system
DAT: dopamine transporter
DCVs: dense core vesicles
ESCRT: endosomal sorting complex required for transport
F-actin: filamentous actin
GABA: gamma-aminobutyric acid
GWAS: genome-wide association studies
GDNF: glial-derived neurotrophic factor
iPSC: induced pluripotent stem cell
LDCVs: large dense-core vesicles
LRRK2: leucine-rich repeat kinase 2
LBs: Lewy bodies
LTP: long-term potentiation
mEPSC: miniature excitatory postsynaptic current
NGF: nerve-growth factor
NSF: N-ethylmaleimide-sensitive factor
ORFs: open reading frames
PD: Parkinson’s disease
PFFs: preformed fibrils
PKA: protein kinase A
PPI: protein–protein interaction
PSD: postsynaptic density
ROS: reactive oxygen species
SPECT: single photon emission computed tomography
SNpc: substantia nigra pars compacta
SNpr: substantia nigra pars reticulata
SV: synaptic vesicle
SVPs: synaptic vesicle precursors
VTA: ventral tegmental area
